# 1,4-Bis(3-methyl­phen­yl)piperazine-2,5-dione

**DOI:** 10.1107/S1600536812026372

**Published:** 2012-06-16

**Authors:** Yongjun Liu, Xuefeng Sun, Yonghong Wen

**Affiliations:** a College of Chemistry and Molecular Engineering, Qingdao University of Science and Technology, Qingdao 266042, People’s Republic of China

## Abstract

The asymmetric unit of the title compound, C_18_H_18_N_2_O_2_, consists of two independent mol­ecules, each of which is located about a center of inversion. The mol­ecules are not planar, showing dihedral angles of 55.84 (9) and 54.10 (8)° between the piperazinedione and the aromatic rings. The piperazine N atoms exhibit a planar configuration. The crystal packing is stabilized by inter­molecular C—H⋯O hydrogen bonds.

## Related literature

For background to the applications of piperazinedione and its derivatives, see: Acharya *et al.* (2001 ▶); Fischer (2003 ▶); Krchnak *et al.* (1996 ▶); Paradisi *et al.* (2002 ▶). For the syntheses and structures of piperazinediones, see: Wen *et al.* (2006 ▶); Zhang *et al.* (2007 ▶).
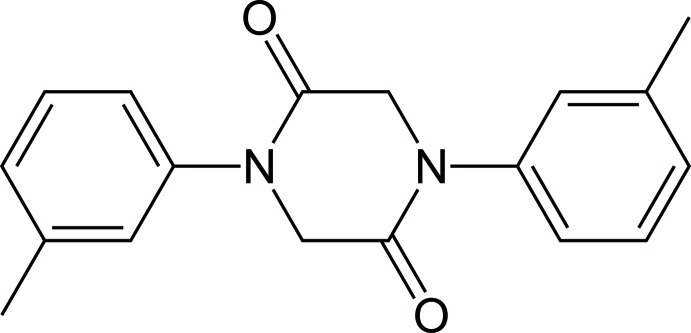


## Experimental

### 

#### Crystal data

C_18_H_18_N_2_O_2_
*M* *_r_* = 294.34
Monoclinic, 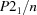

*a* = 12.6608 (15) Å
*b* = 6.1508 (7) Å
*c* = 19.223 (2) Å
β = 95.142 (2)°
*V* = 1490.9 (3) Å^3^
*Z* = 4
Mo *K*α radiation
μ = 0.09 mm^−1^
*T* = 153 K
0.38 × 0.12 × 0.10 mm

#### Data collection

Bruker SMART CCD area-detector diffractometer
Absorption correction: multi-scan (*SADABS*; Sheldrick, 1996 ▶) *T*_min_ = 0.968, *T*_max_ = 0.991
7835 measured reflections
2836 independent reflections
2078 reflections with *I* > 2σ(*I*)
*R*_int_ = 0.029

#### Refinement

*R*[*F* ^2^ > 2σ(*F* ^2^)] = 0.052
*wR*(*F* ^2^) = 0.137
*S* = 1.02
2836 reflections
199 parameters
H-atom parameters constrained
Δρ_max_ = 0.19 e Å^−3^
Δρ_min_ = −0.22 e Å^−3^

### 

Data collection: *SMART* (Bruker, 2001 ▶); cell refinement: *SAINT* (Bruker, 2001 ▶); data reduction: *SAINT*; program(s) used to solve structure: *SHELXTL* (Sheldrick, 2008 ▶); program(s) used to refine structure: *SHELXTL*; molecular graphics: *SHELXTL*; software used to prepare material for publication: *SHELXTL*.

## Supplementary Material

Crystal structure: contains datablock(s) I, global. DOI: 10.1107/S1600536812026372/zq2169sup1.cif

Structure factors: contains datablock(s) I. DOI: 10.1107/S1600536812026372/zq2169Isup2.hkl

Supplementary material file. DOI: 10.1107/S1600536812026372/zq2169Isup3.cml

Additional supplementary materials:  crystallographic information; 3D view; checkCIF report

## Figures and Tables

**Table 1:** Hydrogen-bond geometry (Å, °)

*D*—H⋯*A*	*D*—H	H⋯*A*	*D*⋯*A*	*D*—H⋯*A*
C8—H8*B*⋯O1^i^	0.97	2.53	3.494 (2)	173
C14—H14*A*⋯O2^i^	0.93	2.45	3.325 (3)	158

Symmetry code: (i) 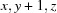.

**Fig. 1.**
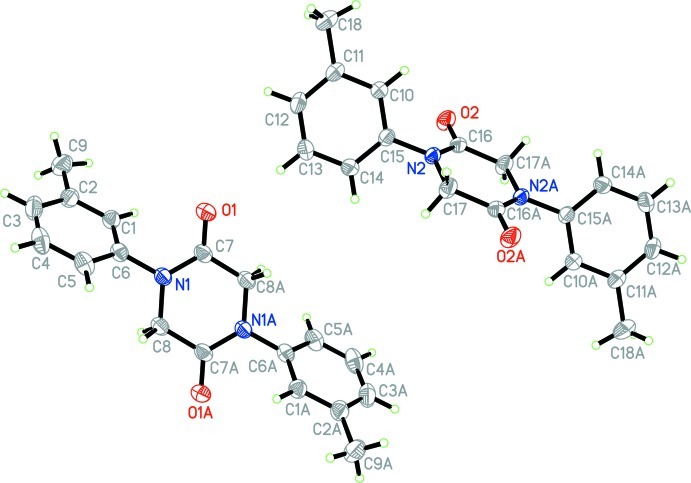
The molecular structure of the title compound, with atom labels and 50% probability displacement ellipsoids.

**Fig. 2.**
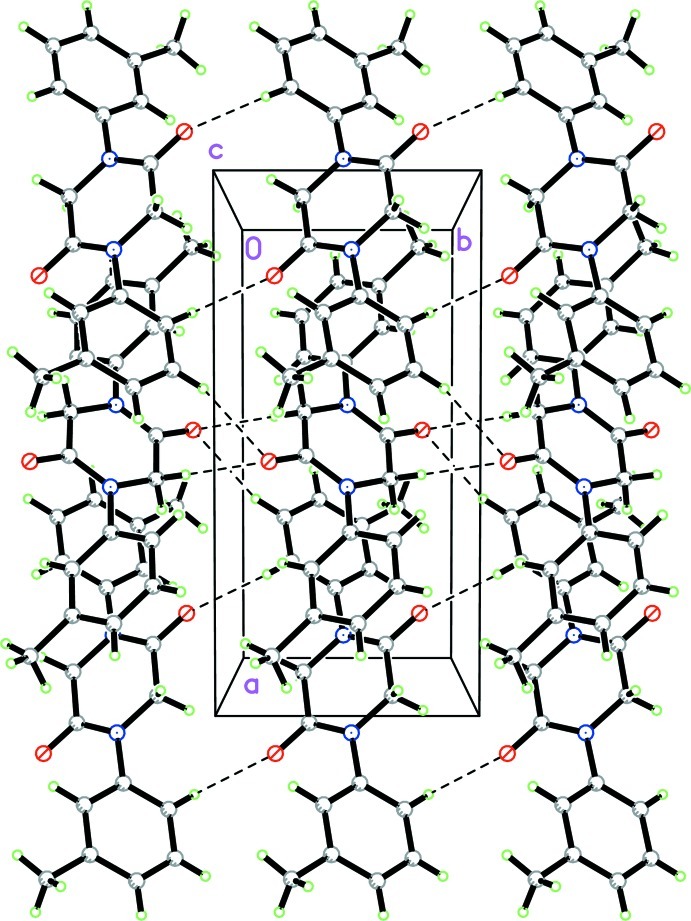
The packing diagram of the title compound, viewed down the c axis, showing the intermolecular hydrogen bonds (dashed lines).

## supplementary crystallographic information

### Comment

Piperazinediones form an important class of biologically active natural products (Fischer *et al.*, 2003), and represent important precursors for the synthesis of peptides and non-natural amino acids (Paradisi *et al.*, 2002). Recently, piperazinediones have gained importance in drug discovery (Krchnak *et al.*, 1996), and opioid receptor agonists and antagonists (Acharya *et al.*, 2001). Here, we report the crystal structure of the title compound.

The title compound consists of two crystallographically independent C_18_H_18_N_2_O_2_ molecules in the asymmetric unit of the centrosymmetric space group *P*2_1_/n (Fig. 1). Each molecule is located on a center of inversion. All bond lengths and angles of two independent molecules are comparable with those in the related compounds (Wen *et al.*, 2006; Zhang *et al.*, 2007). The molecules are not planar showing dihedral angles of 55.84 (9)° and 54.10 (8)° between the piperazinedione and the aromatic rings, which is different from the *ortho*-substituted isomer 1,4-bis(2-methylphenyl)piperazine-2,5-dione (Wen *et al.*, 2006). The sum of the bond angles around N1 (360.00°) and N2 (359.98°) indicates the piperazine N atoms have also a planar configuration, different from the normal pyramidal configuration of the N atom. This difference is mainly due to the π-conjugation effects arising from the presence of the two C=O double bonds. In addition, the crystal packing exhibit intermolecular C—H···O hydrogen bonds (Table 1, Fig. 2).

### Experimental

The title compound was prepared according to the literature method (Wen *et al.*, 2006). Colourless single crystals of the title compound suitable for X-ray diffraction study were obtained by slow evaporation of an ethanol solution over a period of 20 d.

### Refinement

All H atoms were positioned geometricallyand constrained to ride on their parent atoms, with C—H = 0.93 Å and *U*_iso_(H) = 1.2*U*_eq_(C) for aromatic H atoms, with C—H = 0.97 Å and *U*_iso_(H) = 1.2*U*_eq_(C) for methylene H atoms, and with C—H = 0.96 and *U*_iso_(H) = 1.5*U*_eq_(C) for methyl H atoms.

### Crystal data

**Table:**

C_18_H_18_N_2_O_2_	*F*(000) = 624
*M**_r_* = 294.34	*D*_x_ = 1.311 Mg m^−^^3^
Monoclinic, *P*2_1_/*n*	Mo *K*α radiation, λ = 0.71073 Å
Hall symbol: -P 2yn	Cell parameters from 1825 reflections
*a* = 12.6608 (15) Å	θ = 3.2–23.9°
*b* = 6.1508 (7) Å	µ = 0.09 mm^−^^1^
*c* = 19.223 (2) Å	*T* = 153 K
β = 95.142 (2)°	Block, colourless
*V* = 1490.9 (3) Å^3^	0.38 × 0.12 × 0.10 mm
*Z* = 4	

### Data collection

**Table:**

Bruker SMART CCD area-detector diffractometer	2836 independent reflections
Radiation source: fine-focus sealed tube	2078 reflections with *I* > 2σ(*I*)
Graphite monochromator	*R*_int_ = 0.029
phi and ω scans	θ_max_ = 25.7°, θ_min_ = 1.9°
Absorption correction: multi-scan (*SADABS*; Sheldrick, 1996)	*h* = −15→15
*T*_min_ = 0.968, *T*_max_ = 0.991	*k* = −7→7
7835 measured reflections	*l* = −23→12

### Refinement

**Table:**

Refinement on *F*^2^	Primary atom site location: structure-invariant direct methods
Least-squares matrix: full	Secondary atom site location: difference Fourier map
*R*[*F*^2^ > 2σ(*F*^2^)] = 0.052	Hydrogen site location: inferred from neighbouring sites
*wR*(*F*^2^) = 0.137	H-atom parameters constrained
*S* = 1.02	*w* = 1/[σ^2^(*F*_o_^2^) + (0.0664*P*)^2^ + 0.2932*P*] where *P* = (*F*_o_^2^ + 2*F*_c_^2^)/3
2836 reflections	(Δ/σ)_max_ < 0.001
199 parameters	Δρ_max_ = 0.19 e Å^−^^3^
0 restraints	Δρ_min_ = −0.22 e Å^−^^3^

### Special details

**Table:**

Geometry. All e.s.d.'s (except the e.s.d. in the dihedral angle between two l.s. planes) are estimated using the full covariance matrix. The cell e.s.d.'s are taken into account individually in the estimation of e.s.d.'s in distances, angles and torsion angles; correlations between e.s.d.'s in cell parameters are only used when they are defined by crystal symmetry. An approximate (isotropic) treatment of cell e.s.d.'s is used for estimating e.s.d.'s involving l.s. planes.
Refinement. Refinement of *F*^2^ against ALL reflections. The weighted *R*-factor *wR* and goodness of fit *S* are based on *F*^2^, conventional *R*-factors *R* are based on *F*, with *F* set to zero for negative *F*^2^. The threshold expression of *F*^2^ > σ(*F*^2^) is used only for calculating *R*-factors(gt) *etc*. and is not relevant to the choice of reflections for refinement. *R*-factors based on *F*^2^ are statistically about twice as large as those based on *F*, and *R*- factors based on ALL data will be even larger.

### Fractional atomic coordinates and isotropic or equivalent isotropic displacement parameters (Å^2^)

**Table:**

	*x*	*y*	*z*	*U*_iso_*/*U*_eq_	
O2	0.14816 (10)	0.1901 (2)	0.50117 (7)	0.0517 (4)	
N2	0.09833 (10)	0.5171 (2)	0.54147 (8)	0.0372 (4)	
O1	0.53271 (11)	1.1732 (2)	0.59161 (8)	0.0571 (4)	
N1	0.58560 (11)	1.5000 (2)	0.55280 (8)	0.0423 (4)	
C15	0.19661 (13)	0.5530 (3)	0.58423 (9)	0.0369 (4)	
C16	0.08291 (13)	0.3358 (3)	0.50265 (9)	0.0383 (4)	
C14	0.25272 (14)	0.7430 (3)	0.57627 (10)	0.0432 (5)	
H14A	0.2275	0.8470	0.5438	0.052*	
C6	0.67677 (14)	1.5123 (3)	0.60368 (10)	0.0434 (5)	
C7	0.52080 (14)	1.3255 (3)	0.55088 (11)	0.0431 (5)	
C1	0.75220 (14)	1.3485 (3)	0.60645 (10)	0.0442 (5)	
H1A	0.7416	1.2280	0.5774	0.053*	
C10	0.23305 (13)	0.4011 (3)	0.63376 (9)	0.0410 (5)	
H10A	0.1942	0.2748	0.6391	0.049*	
C13	0.34654 (15)	0.7763 (3)	0.61707 (11)	0.0499 (5)	
H13A	0.3853	0.9026	0.6116	0.060*	
C8	0.56977 (15)	1.6836 (3)	0.50490 (11)	0.0487 (5)	
H8A	0.6345	1.7026	0.4821	0.058*	
H8B	0.5608	1.8130	0.5326	0.058*	
C17	0.02008 (14)	0.6913 (3)	0.54148 (11)	0.0480 (5)	
H17A	0.0532	0.8236	0.5267	0.058*	
H17B	0.0038	0.7134	0.5893	0.058*	
C12	0.38306 (15)	0.6248 (3)	0.66558 (11)	0.0505 (5)	
H12A	0.4468	0.6493	0.6924	0.061*	
C11	0.32667 (14)	0.4345 (3)	0.67562 (10)	0.0443 (5)	
C5	0.69087 (17)	1.6911 (3)	0.64691 (11)	0.0563 (6)	
H5A	0.6405	1.8015	0.6450	0.068*	
C9	0.92602 (17)	1.1862 (4)	0.65370 (13)	0.0715 (7)	
H9A	0.9037	1.0751	0.6205	0.107*	
H9B	0.9353	1.1243	0.6997	0.107*	
H9C	0.9920	1.2472	0.6420	0.107*	
C4	0.78057 (19)	1.7042 (4)	0.69301 (12)	0.0656 (7)	
H4A	0.7901	1.8230	0.7229	0.079*	
C2	0.84310 (15)	1.3621 (4)	0.65189 (10)	0.0507 (5)	
C18	0.36529 (18)	0.2729 (4)	0.73046 (12)	0.0676 (7)	
H18A	0.3167	0.1528	0.7298	0.101*	
H18B	0.3697	0.3412	0.7755	0.101*	
H18C	0.4341	0.2210	0.7211	0.101*	
C3	0.85602 (18)	1.5430 (4)	0.69520 (12)	0.0637 (6)	
H3A	0.9166	1.5552	0.7261	0.076*	

### Atomic displacement parameters (Å^2^)

**Table:**

	*U*^11^	*U*^22^	*U*^33^	*U*^12^	*U*^13^	*U*^23^
O2	0.0451 (8)	0.0387 (7)	0.0687 (9)	0.0118 (6)	−0.0098 (7)	−0.0062 (7)
N2	0.0339 (8)	0.0303 (8)	0.0456 (9)	0.0019 (6)	−0.0069 (7)	0.0003 (7)
O1	0.0545 (8)	0.0430 (8)	0.0724 (10)	−0.0054 (6)	−0.0018 (7)	0.0148 (7)
N1	0.0392 (8)	0.0338 (8)	0.0535 (10)	−0.0026 (6)	0.0027 (7)	0.0021 (7)
C15	0.0342 (9)	0.0362 (9)	0.0392 (10)	0.0025 (7)	−0.0029 (8)	−0.0016 (8)
C16	0.0383 (9)	0.0311 (9)	0.0445 (11)	0.0019 (7)	−0.0019 (8)	0.0034 (8)
C14	0.0472 (11)	0.0374 (10)	0.0439 (10)	−0.0030 (8)	−0.0015 (9)	0.0029 (9)
C6	0.0432 (10)	0.0423 (10)	0.0455 (11)	−0.0072 (8)	0.0087 (9)	0.0010 (9)
C7	0.0400 (10)	0.0313 (9)	0.0592 (12)	0.0005 (8)	0.0102 (9)	0.0007 (9)
C1	0.0455 (11)	0.0457 (11)	0.0413 (11)	−0.0040 (9)	0.0031 (9)	−0.0003 (9)
C10	0.0378 (10)	0.0401 (10)	0.0441 (10)	−0.0011 (8)	−0.0018 (9)	0.0047 (9)
C13	0.0447 (11)	0.0495 (11)	0.0544 (12)	−0.0134 (9)	−0.0009 (10)	−0.0011 (10)
C8	0.0428 (10)	0.0357 (10)	0.0672 (14)	−0.0047 (8)	0.0027 (10)	0.0067 (10)
C17	0.0431 (10)	0.0355 (10)	0.0621 (13)	0.0075 (8)	−0.0134 (10)	−0.0099 (9)
C12	0.0364 (10)	0.0625 (13)	0.0506 (12)	−0.0052 (9)	−0.0078 (9)	−0.0038 (10)
C11	0.0387 (10)	0.0530 (12)	0.0401 (10)	0.0060 (9)	−0.0025 (8)	0.0015 (9)
C5	0.0569 (13)	0.0472 (12)	0.0662 (14)	−0.0086 (10)	0.0129 (11)	−0.0103 (11)
C9	0.0516 (13)	0.0829 (17)	0.0775 (17)	0.0030 (12)	−0.0078 (12)	0.0164 (14)
C4	0.0691 (15)	0.0647 (15)	0.0635 (15)	−0.0252 (12)	0.0093 (13)	−0.0206 (12)
C2	0.0451 (11)	0.0616 (13)	0.0454 (11)	−0.0088 (10)	0.0037 (9)	0.0078 (10)
C18	0.0633 (14)	0.0744 (15)	0.0606 (14)	0.0066 (12)	−0.0191 (12)	0.0146 (13)
C3	0.0527 (13)	0.0801 (17)	0.0565 (13)	−0.0237 (12)	−0.0040 (11)	−0.0001 (13)

### Geometric parameters (Å, º)

**Table:**

O2—C16	1.221 (2)	C8—C7^ii^	1.499 (3)
N2—C16	1.346 (2)	C8—H8A	0.9700
N2—C15	1.445 (2)	C8—H8B	0.9700
N2—C17	1.459 (2)	C17—C16^i^	1.500 (2)
O1—C7	1.221 (2)	C17—H17A	0.9700
N1—C7	1.349 (2)	C17—H17B	0.9700
N1—C6	1.446 (2)	C12—C11	1.393 (3)
N1—C8	1.460 (2)	C12—H12A	0.9300
C15—C10	1.383 (2)	C11—C18	1.499 (3)
C15—C14	1.383 (2)	C5—C4	1.379 (3)
C16—C17^i^	1.500 (2)	C5—H5A	0.9300
C14—C13	1.379 (3)	C9—C2	1.505 (3)
C14—H14A	0.9300	C9—H9A	0.9600
C6—C5	1.380 (3)	C9—H9B	0.9600
C6—C1	1.386 (3)	C9—H9C	0.9600
C7—C8^ii^	1.499 (3)	C4—C3	1.375 (3)
C1—C2	1.384 (3)	C4—H4A	0.9300
C1—H1A	0.9300	C2—C3	1.391 (3)
C10—C11	1.387 (2)	C18—H18A	0.9600
C10—H10A	0.9300	C18—H18B	0.9600
C13—C12	1.369 (3)	C18—H18C	0.9600
C13—H13A	0.9300	C3—H3A	0.9300
			
C16—N2—C15	121.08 (14)	N2—C17—C16^i^	118.32 (15)
C16—N2—C17	122.92 (14)	N2—C17—H17A	107.7
C15—N2—C17	115.97 (14)	C16^i^—C17—H17A	107.7
C7—N1—C6	120.45 (16)	N2—C17—H17B	107.7
C7—N1—C8	123.32 (15)	C16^i^—C17—H17B	107.7
C6—N1—C8	116.22 (14)	H17A—C17—H17B	107.1
C10—C15—C14	120.26 (16)	C13—C12—C11	121.34 (17)
C10—C15—N2	120.36 (15)	C13—C12—H12A	119.3
C14—C15—N2	119.37 (15)	C11—C12—H12A	119.3
O2—C16—N2	123.79 (16)	C10—C11—C12	117.77 (17)
O2—C16—C17^i^	117.46 (16)	C10—C11—C18	121.18 (18)
N2—C16—C17^i^	118.75 (15)	C12—C11—C18	121.04 (17)
C13—C14—C15	119.16 (17)	C4—C5—C6	119.2 (2)
C13—C14—H14A	120.4	C4—C5—H5A	120.4
C15—C14—H14A	120.4	C6—C5—H5A	120.4
C5—C6—C1	120.31 (18)	C2—C9—H9A	109.5
C5—C6—N1	120.09 (18)	C2—C9—H9B	109.5
C1—C6—N1	119.54 (16)	H9A—C9—H9B	109.5
O1—C7—N1	123.53 (18)	C2—C9—H9C	109.5
O1—C7—C8^ii^	118.20 (16)	H9A—C9—H9C	109.5
N1—C7—C8^ii^	118.26 (17)	H9B—C9—H9C	109.5
C2—C1—C6	120.76 (18)	C3—C4—C5	120.5 (2)
C2—C1—H1A	119.6	C3—C4—H4A	119.7
C6—C1—H1A	119.6	C5—C4—H4A	119.7
C15—C10—C11	120.95 (17)	C1—C2—C3	118.2 (2)
C15—C10—H10A	119.5	C1—C2—C9	120.7 (2)
C11—C10—H10A	119.5	C3—C2—C9	121.12 (19)
C12—C13—C14	120.50 (18)	C11—C18—H18A	109.5
C12—C13—H13A	119.8	C11—C18—H18B	109.5
C14—C13—H13A	119.8	H18A—C18—H18B	109.5
N1—C8—C7^ii^	118.38 (15)	C11—C18—H18C	109.5
N1—C8—H8A	107.7	H18A—C18—H18C	109.5
C7^ii^—C8—H8A	107.7	H18B—C18—H18C	109.5
N1—C8—H8B	107.7	C4—C3—C2	121.0 (2)
C7^ii^—C8—H8B	107.7	C4—C3—H3A	119.5
H8A—C8—H8B	107.1	C2—C3—H3A	119.5
			
C16—N2—C15—C10	−55.7 (2)	C14—C15—C10—C11	−0.8 (3)
C17—N2—C15—C10	126.27 (19)	N2—C15—C10—C11	−179.35 (17)
C16—N2—C15—C14	125.74 (19)	C15—C14—C13—C12	−0.9 (3)
C17—N2—C15—C14	−52.3 (2)	C7—N1—C8—C7^ii^	−2.2 (3)
C15—N2—C16—O2	1.0 (3)	C6—N1—C8—C7^ii^	177.36 (16)
C17—N2—C16—O2	178.81 (19)	C16—N2—C17—C16^i^	1.3 (3)
C15—N2—C16—C17^i^	−179.17 (17)	C15—N2—C17—C16^i^	179.26 (16)
C17—N2—C16—C17^i^	−1.3 (3)	C14—C13—C12—C11	−0.5 (3)
C10—C15—C14—C13	1.6 (3)	C15—C10—C11—C12	−0.6 (3)
N2—C15—C14—C13	−179.86 (17)	C15—C10—C11—C18	178.63 (19)
C7—N1—C6—C5	−126.3 (2)	C13—C12—C11—C10	1.3 (3)
C8—N1—C6—C5	54.1 (2)	C13—C12—C11—C18	−177.93 (19)
C7—N1—C6—C1	56.7 (2)	C1—C6—C5—C4	−0.3 (3)
C8—N1—C6—C1	−122.93 (19)	N1—C6—C5—C4	−177.27 (19)
C6—N1—C7—O1	1.7 (3)	C6—C5—C4—C3	1.1 (3)
C8—N1—C7—O1	−178.69 (18)	C6—C1—C2—C3	0.8 (3)
C6—N1—C7—C8^ii^	−177.35 (17)	C6—C1—C2—C9	−178.7 (2)
C8—N1—C7—C8^ii^	2.2 (3)	C5—C4—C3—C2	−0.9 (4)
C5—C6—C1—C2	−0.7 (3)	C1—C2—C3—C4	0.0 (3)
N1—C6—C1—C2	176.35 (17)	C9—C2—C3—C4	179.4 (2)

Symmetry codes: (i) −*x*, −*y*+1, −*z*+1; (ii) −*x*+1, −*y*+3, −*z*+1.

### Hydrogen-bond geometry (Å, º)

**Table:**

*D*—H···*A*	*D*—H	H···*A*	*D*···*A*	*D*—H···*A*
C8—H8*B*···O1^iii^	0.97	2.53	3.494 (2)	173
C14—H14*A*···O2^iii^	0.93	2.45	3.325 (3)	158

Symmetry code: (iii) *x*, *y*+1, *z*.
